# Bloodthirsty bites: host-feeding patterns of phlebotomine sand flies from two localities in the Aegean Region of Türkiye

**DOI:** 10.1186/s12917-025-04881-y

**Published:** 2025-07-25

**Authors:** Metin Pekagirbas, Fatma Bursali, Serkan Bakirci

**Affiliations:** 1 Faculty of Veterinary Medicine, Department of Parasitology, Aydın Adnan Menderes University, Aydin, Türkiye; 2 Faculty of Science, Department of Biology, Aydın Adnan Menderes University, Aydin, Türkiye; 3 Institue of Infection, Veterinary and Ecological Sciences, University of Liverpool, Liverpool, UK

**Keywords:** Blood-meal, ELISA, PCR, Feeding patterns, Sand fly, Türkiye

## Abstract

**Background:**

This study determined the blood-feeding patterns of different wild-caught sandflies collected from Aydin and Mugla provinces located in the Aegean region, Türkiye. Adult sand fly specimens (194 females and 86 males, 280 in total) were collected from two different villages using three CDC light traps in August and October 2024. Of the 194 female specimens, 38 were found to be blood-fed, 137 were non-blood-fed, and 19 were gravid. Among the blood fed, 23 specimens comprising 20 *Phlebotomus papatasi* and 3 *P. tobbi* were from Aydin and 15 specimens comprising 14 *P. major* s.l., 1 *P. alexandri* were from Mugla. Blood-feeding patterns of these species was determined using direct ELISA technique and multiplex PCR method.

**Results:**

All the samples collected from Aydin blood fed from *Bos taurus* whereas females sampled from Mugla locality blood-fed from *Canis lupus* (8/15) and *Gallus domesticus* (7/15). These findings validated using both PCR and ELISA. Although both methods commonly used in blood meal analysis have inherent limitations and disadvantages, all samples in this study were successfully analyzed, and the results from both methods showed a high level of agreement. The efficacy of the methods was compared using McNemar's test.

**Conclusion:**

To the best of our knowledge, this is the first study in the region to compare ELISA and PCR methodologies in determining the host feeding patterns of sand flies. The detection of blood meal in field-caught sand flies has the potential to facilitate a more comprehensive understanding of the eco-epidemiology of vector-borne diseases, thereby contributing to the planning of strategic control methods.

**Supplementary Information:**

The online version contains supplementary material available at 10.1186/s12917-025-04881-y.

## Background

Phlebotomine sand fly species are considered to be significant vectors for various pathogens, including protozoa (e.g. *Leishmania* spp.), bacteria (e.g. *Bartonella* sp.), and phleboviruses [43]. Of the 1,000 known species of sandflies, a small percentage (10%) belonging to the genera *Phlebotomus* Rondani & Berte, 1840, *Sergentomyia* Franca & Parrot, 1920, and *Lutzomyia* França, 1924 act as vectors of the disease. They are a nuisance both for the diseases they transmit and for the pain caused by their bites [12, 13, 59]. Leishmaniasis, a protozoan disease with cutaneous, visceral, mucocutaneous forms, carries a high global burden, particularly in regions such as Brazil, Bangladesh, Ethiopia, Sudan, Afghanistan, Ethiopia, Iran, Syria, and Türkiye [2, 48, 52]. Sandflies also transmit phleboviruses, which cause fever, nausea, vomiting, and neurological complications [1].

Türkiye is endemic for leishmaniasis, with *Leishmania donovani*/*infantum* and *Leishmania tropica* causing visceral, cutaneous and canine leishmaniasis [30]. To date, four *Leishmania* species (*L. infantum*, *L. tropica*, *L. major*, *L. donovani*) have been reported, with *L. infantum* being the most widely distributed [48]. Visceraland canine leishmaniasis cases are primarily reported from the Aegean and Mediterranean regions, whereas cutaneous leishmaniasis cases are predominantly found in the Eastern and Southeastern regions [25, 46]. Studies in the Aegean region, encompassing Muğla and Aydın, have reported the presence of *L. infantum* in both human and canine samples. Furthermore, investigations in this area have revealed that *L. tropica* and *L. major* are the causative agents of cutaneous leishmaniasis in humans [22, 48, 58]. Türkiye is home to 28 sand fly species including 24 *Phlebotomus* and 4 *Sergentomyia* species [21]. Previous faunal studies within the study area identified nine *Phlebotomus* and three *Sergentomyia* in Aydın [3], with* Phlebotomus* (*Larroussius*) *major* s.l. (Annandale, 1910) and *Phlebotomus*(*Larroussius*) *tobbi* (Adler, Theodor& Lourie, 1930), *Phlebotomus* (*Phlebotomus*) *papatasi* (Scopoli, 1786) and *Phlebotomus* (*Phlebotomus*) *sergenti* (Parrot, 1917) recognized as potential leishmaniasis vectors [3]. Similarly, 12 *Phlebotomus* and three *Sergentomyia* are distributed in Mugla [49] and *P. major* s.l. and *P. tobbi* are identified as the most common species and potential vectors for leishmaniasis in the region [49].

Understanding the blood-feeding patterns of hematophagous insects is particularly important in public health, as it shows the frequency with which they feed on vertebrate hosts. The blood meals of these insects, such as sand flies and mosquitoes, can provide valuable insights into their host-feeding preferences and patterns. This information is crucial for understanding the transmission dynamics of vector-borne diseases, highlighting the paramount ecological and epidemiological importance of a comprehensive understanding of insect blood-feeding patterns [10, 11, 40].

Sand flies typically feed regularly, at intervals of 3–6 days, to obtain nutrients they require to provide protein for egg development [15, 30, 41]. As demonstrated by several studies that have analyzed the blood meals of captured sand flies using molecular and biochemical techniques, sand flies exhibit a remarkable diversity in their feeding preferences [5, 15, 28, 29, 63]. Host preferences vary widely, from highly specific to opportunistic, both between different geographical regions and even within the same species. Old World sand flies belonging to the genera *Phlebotomus* and *Sergentomyia* have been observed to exhibit zoophilic and anthropophilic feeding tendencies, depending on the specific species [14, 38, 54, 57]. Furthermore, sand flies have been observed to consume a mixture of blood from different hosts [5, 24, 61] . While sand flies exhibit a certain degree of phenotypic plasticity in their feeding behavior, they are also influenced by environmental factors and the availability of resources [12]. Furthermore, the type of habitat (e.g. forest, savannah) influences the availability of potential blood sources and thus, feeding patterns [12].

To date, different methodologies have been utilized to analyze blood samples from sand flies, which have been observed feeding on a variety of hosts [44]. In recent years, several molecular techniques such as quantitative polymerase chain reaction (qPCR), and multiplex PCR have been frequently used [50]. These techniques, targeting cytochrome b and cytochrome oxidase c genes, are complemented by expensive and time-consuming methods such as DNA sequence analysis [30, 34, 62]. In addition, the MALDI-TOF method for protein profiling is characterized by its high sensitivity. However, it is important to note that this method is associated with significant financial costs and requires expertise for its successful implementation [26]. In contrast, serology-based techniques, including precipitation and enzyme-linked immunosorbent assay (ELISA), continue to be utilized with success in contemporary clinical practice due to their cost-effectiveness when compared to alternative methods [23].

The specific aim of this study is to elucidate the host-feeding patterns of sand flies from the collected localities in the Aegean region of Türkiye using PCR and ELISA methods.

## Material and methods

### Sand fly collection and morphological identification

The study area included Gokceovacik village (36° 80′15’’N; 28°97′68’’E) in the Dalaman district of Mugla Province and Asagikayacik village (37° 88′46’’N; 27°94′05’E) in the inner parts of the province of Aydin. These areas were selected based on previous studies of zoonotic visceral leishmaniasis (ZVL), cutaneous leishmaniasis (CL) in human, and canine leishmaniasis (CanL) in dogs [22, 45, 48]. Adult sand flies were collected using three CDC light traps (John W. Hock, Gainesville, FL, USA) per village in August and October 2024 (one night x per village x two house x three traps). The light traps were set near the walls, at a height of 1.5 m from the ground. The trap locations were determined based on possible breeding and resting sites of sand flies, such as animal barns, chicken nests, etc. The traps were set at 18:00 pm and collected at 06:00 am. The captured specimens counted and stored in Eppendorf tubes in liquid nitrogen for subsequent morphological identification. Blood-fed female specimens were separated and dissected under a stereo microscope. The dissected parts were then mounted on microscope slides for morphological identification using established identification keys [4, 35, 37]. The thorax and abdomen of the female sandflies were stored in Eppendorf tubes for blood meal analysis prior to DNA isolation.

### Blood feeding pattern determination

Individual sand flies were homogenized in 150 µl phosphate-buffered saline (PBS). 50 µl was used for DNA isolation, while the remainder was used to determine host preference using the ELISA method. DNA was extracted from 38 individual blood-fed sand flies using an Invitrogen PureLink genomic DNA isolation kit. To determine the blood-feeding patterns of these insects, the mitochondrial cytochrome b (*cytb*) gene region was amplified via multiplex PCR using specific primers [33, 36, 51]. PCR reactions were conducted under standard conditions, with a final reaction volume of 25 μl containing 50 ng DNA, 2X 12.5 μl Master mix, 0.25 μl each of primers (Human741F-GGCTTACTTCTCTTCATTCTCTCCT; Dog368F-GGAATTGTACTATTATTCCGCAACCA; Cow121F-CATCGGCACAAATTTAGTCG; HorseF- CCCTACATCGGTACTACCC; Goat894F-CCTAATCTTAGTACTTGTACCCTTCCTCT; ChickenF-CCCCTCAGAATGATATTTGTCCTCA ChickenR- CCATCCAACATCTCAGCATGATGAAA; UNREV1025- GGTTGTCCTCCAATTCATGTTA) and 10.25 μl ddH_2_0. The PCR reaction was carried out with an initial denaturation at 95 °C for 5 min, 40 cycles of denaturation at 95 °C for 30 s, annealing at 57 °C for 1 min, extension at 72 °C for 1 min, then final extension: 72 °C for 5 min. Negative control (distilled water) and positive controls (different host bloods) were included to monitor contamination and accuracy. Amplified PCR products were visualized on 2% agarose gel to confirm successful amplification. The band sizes for potential hosts were as follows: *Homo sapiens* (334 bp), *Capra hircus* (132 bp), *Canis lupus* (680 bp), *Bos taurus* (561 bp), *Equus caballus* (500 bp), and *Gallus domesticus* (383 bp) [11].

Also, direct ELISA technique was used to confirm blood feeding patterns and to determine the host preference of the samples that were negative in PCR reaction [6]. The method relies on the reaction between antibodies specific to potential host blood (human, cow, dog, horse, chicken) and the ingested blood meal within the sand fly. Homogenates in PBS were subjected to an ELISA to detect specific antibodies against various host species according to [10]. Wells of a microplate (Corning 96-well Clear Round Bottom Polystyrene Not Treated Sterile Microplate) were coated with antibodies (anti-human IgG, anti-horse IgG, anti-bovine IgG, anti-dog IgG, and anti-chicken IgG) specific to various host species. These five antibodies were used to identify potential blood meal sources. Peroxidase-conjugated anti-host IgG antibody was added to the wells and incubated. After washing, ABTS substrate was added, resulting in a colorimetric reaction at 405 nm using a microplate reader (BioTek ELx808 Absorbance Plate Reader). The resulting color changes were indicative of the presence of specific host blood. Negative controls were represented by unfed sand flies and were utilized to determine the cut-off value for the positive identification of each blood source. Positive controls, consisting of blood from each potential host species, were included for validation purposes. The choice of antibodies was based on the prevalence of these host species in the study region.

### Statistical analysis

McNemar's test was employed to compare the efficacy of the PCR and ELISA methods in blood feeding pattern determination (*p* > 0.05). The data analysis were performed using SPSS (v29). Comparisons were only conducted when discordant pairs were present; no analysis was performed when both methods yielded either 100% or 0% detection.

## Results

### Blood feeding pattern determination

Two hundred eighty sand fly specimens (194 females and 86 males, F/M ratio: 2,25) were collected from two different localities during the study. Most of the specimens were identified as *P. major* s.l (210/280, %75) and *P. tobbi* (31/280, %11) in the study area. Of the 194 female specimens, 23 from Aydin and 15 from Mugla were found to be blood-fed (38), 137 were non-blood-fed, and 19 were gravid. Following the dissection and identification of blood fed sand flies, four (*P. major* s.l, *P. alexandri*, *P. tobbi*, *P. sergenti*) and three (*P. papatasi*, *P. major* s.l, *P. tobbi*) *Phlebotomus* species were found in Mugla and Aydin, respectively (Table 1).

**Table 1:** The total number of collected sand fly species

Sand fly Species	Locality	
	Aydın	Muğla
*P. major* s.l	62 (47.6%)	148 (83.6%)
*P. papatasi*	31 (30.09%)	-
*P. alexandri*	-	19 (10.7%)
*P. tobbi*	10 (9.7%)	8 (4.51%)
*P. sergenti*	-	2 (1.12%)
Total number of sand flies	103	177

**Fig. 1:**
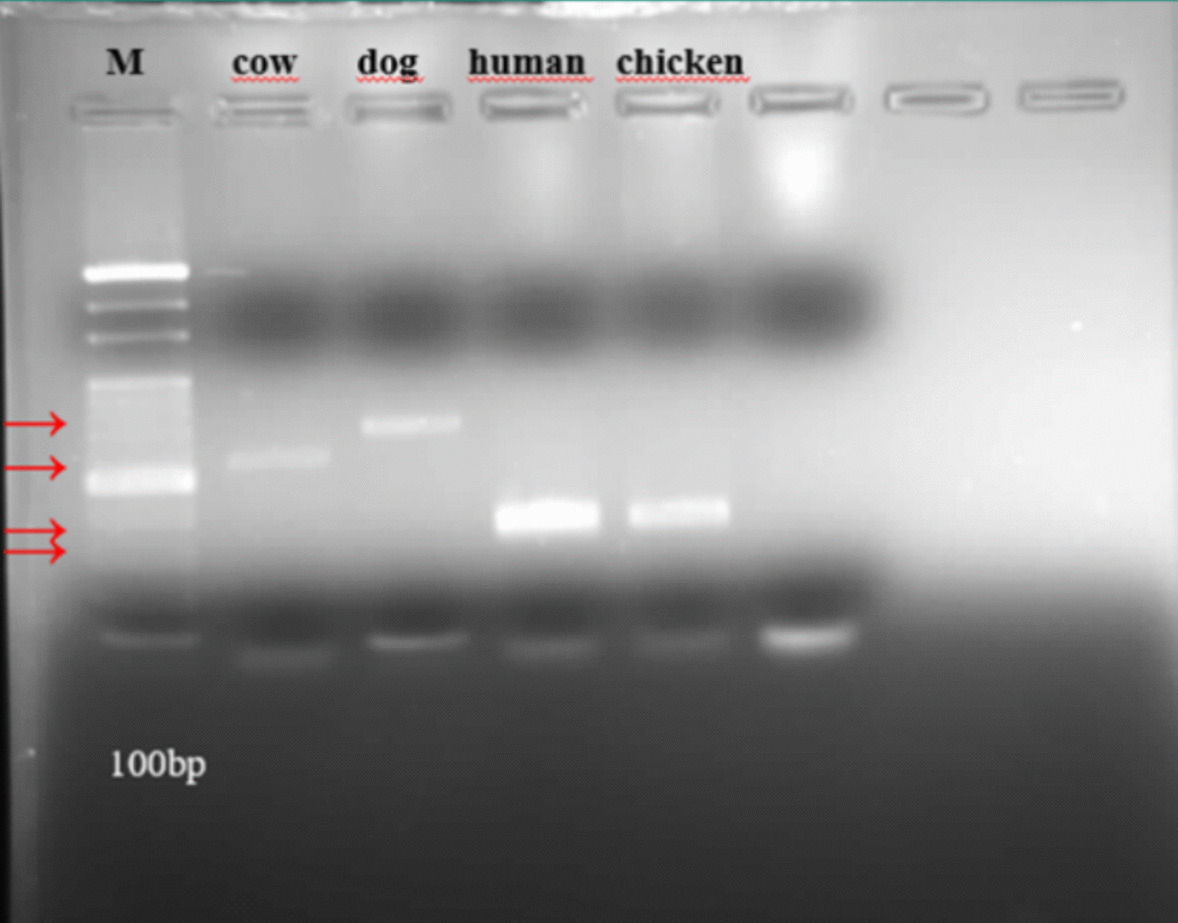
Agarose-gel image of possible host’s PCR products (M: marker (GeneRuler 100 bp DNA Ladder) band size for *Bos taurus*: 561 bp *Canis lupus*: 680 bp *Homo sapiens*: 334 bp *Gallus domesticus*: 383 bp)

**Fig. 2:**
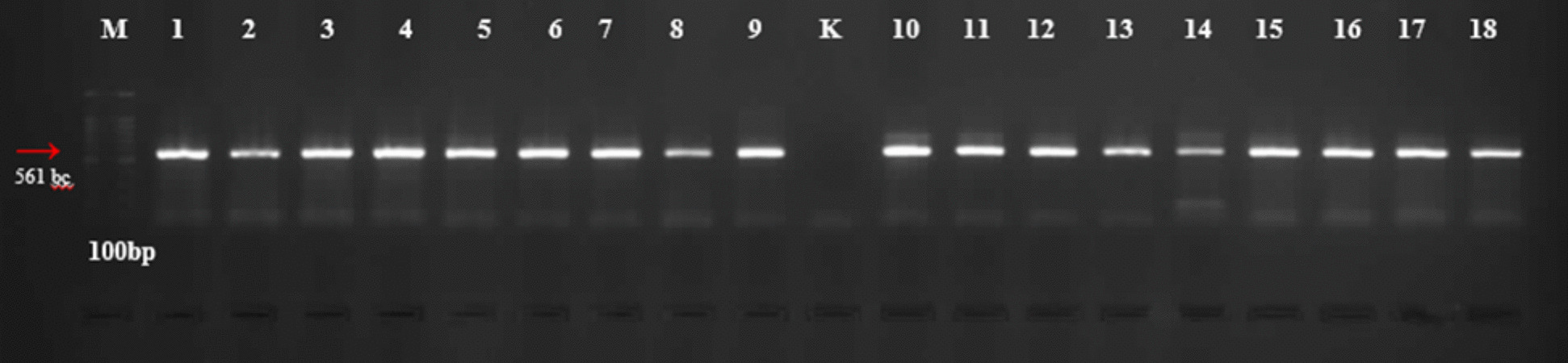
Agarose-gel image of PCR products from Aydin (M: GeneRuler 100 bp DNA Ladder, 1-18 *Bos taurus* PCR products)

In total, DNA from 38 sand flies was utilized for the amplification of the *cyt b* gene region to ascertain their feeding patterns, and the results of the PCR showed that sand flies females in the sampling locations predominantly fed on *Bos taurus* (60.5%), followed by *Canis lupus* (21%) and *Gallus domesticus* (18.4%), respectively (Fig. 1). All the 23 females sampled from Aydin (20 *Phlebotomus papatasi*, three *P. tobbi*) fed on *Bos taurus*. In contrast, of the 15 female sand flies (14 *P. major* s.l., one *P. alexandri*) sampled from Mugla, eight fed on dog blood and seven on *Gallus domesticus* blood (Fig. 2, Tables 2 and 3).

**Table 2:** Natural blood-feeding pattern of sand flies collected from Aydin and Mugla provinces in Türkiye

Locality	Number of blood fed sand flies	Method	Cow	Dog	Chicken	Human	Horse
Aydin	23	PCR	23/23 (100%)	0/23	0/23	0/23	0/23
Aydin		ELISA	23/23 (100%)	0/23	0/23	0/23	0/23
Mugla	15	PCR	0/15	8/15 (53%)	0/15	0/15	0/15
Mugla		ELISA	0/15	8/15 (53%)	7/15 (47%)	0/15	0/15
Overall percentage			60.52%	21.05%	18.42%	0%	0%

**Table 3:** The host preference of the identified blood fed sand fly species

Sandfly species	Locality	Host Blood Sources				
		Cow	Dog	Chicken	Human	Horse
*P. papatasi*	Aydin	20	-	-	-	-
*P. tobbi*	Aydin	3	-	-	-	-
*P. major* s.l	Mugla	-	7	7		-
*P. alexandri*	Mugla	-	1	-	-	-
Total number of sand flies		23	8	7		

The blood meal pattern sand fly specimens were also validated by ELISA. In all localities, cows were the most common hosts, followed by dog and chicken hosts. Most of the blood meals were derived from a single vertebrate host among the five tested species (human or animal). A significant portion of the traps were installed inside barns, and each household typically maintains a dog and a small chicken coop. Consequently, the findings closely reflect the conditions of the areas where the traps were deployed (Tables 1, 2 and 3). McNemar's test was employed to compare the efficacy of the methods. While both PCR and ELISA demonstrated 100% detection for cow samples from Aydin and dog samples from Mugla, a statistically significant difference was observed in the detection rates for chicken samples from Mugla (*p* = 0.016).

## Discussion

This study determined the natural feeding patterns of sand fly populations from the Aegean region of Türkiye using the PCR and ELISA methods. A total of 38 blood-fed sand flies were collected from various locations between August and October 2024. Sand fly specimens were analyzed to identify the blood source of these wild-type populations, and the results showed that cow blood was the host species of all the Aydin sand fly species. In addition, dog and chicken blood meals were identified as the host species of the Mugla specimens, albeit at much lower frequencies compared to the preference ratio for cow blood. Mixed blood meals from two different hosts were not observed. The present study, which was conducted in a single location in both study areas, has certain limitations. It is evident that, despite the species identified in this study being comparable to those identified in previous large-scale fauna studies conducted in the region, the species diversity in this study is comparatively lower [3, 49]. 

Sand flies have been demonstrated to display a broad spectrum of host preferences, encompassing humans, animals, and even ectothermic vertebrates such as amphibians [12, 17, 20, 28]. The blood-feeding patterns of these insects can be influenced by the availability of potential hosts as well as local environmental conditions, host body size and attractiveness [12]. Numerous species are opportunistic feeders, acquiring blood meals from accessible hosts irrespective of species. For example, *P. perniciosus* from Spain, Italy and Portugal displayed opportunistic feeding habits, with no clear host preference [8, 18, 39]. Azmi et al. [5] found that *P. papatasi* and *P. sergenti* in Palestine fed on humans, hyraxes, rats, livestock, and birds, with some individuals even taking mixed blood meals. Jaouadi et al. [27] reported that sand flies in Tunisia primarily fed on humans, rodents, and livestock. Salah et al. [53] found that *P. sergenti* in Palestine fed on humans, livestock, birds, and dogs. A small percentage of sand flies had mixed blood meals from both avian and mammalian sources. At the species level, chicken blood was the most frequently detected avian source. Human, cow, and dog blood were also identified among the mammalian meals. In contrast to the findings of other studies on the *P. papatasi*, which were previously described as opportunistic [56] and generally reported to be highly anthropophilic [9], human blood was not identified in this study. Although this situation is interesting for the *P. papatasi* with high anthropophilic characteristics, it is thought that it may be due to its opportunistic character and the fact that the animal shelters are located very close to the houses in the locations used in the study and other mammalian hosts in the environment.

In Türkiye*, Phlebotomus major* s.l., recognized as the vector of *L. infantum*, is a complex belonging to the *Larrouissius* subspecies and has been documented as the dominant species in both study areas in previous publications [3, 32, 49]. While *P. major* s.l. was not detected among the blood fed specimens in Aydın, *P. papatasi*, which is the main vector for *L. major* [31], was identified in the highest number. In Muğla *P. major* s.l. was found in the highest number among the bloodfed specimen. Despite the large number of studies showing these species as potential or definite vectors for *Leishmania*, the literature on the feeding habits of sand fly species in Türkiye is quite limited. Karakuş et al. [30] found that the most common food preference of sand flies was dog blood, followed by humans, mice, cats, and cows in terms of frequency in their study in Aydin. The same study revealed that the *P. neglectus* showed a clear preference for human blood. This finding was later shown by Ozbel et al. [47] to be the most common blood-feeding preference (80%) among the *P. tobbi* species. Consistent with the results of the present study, Yetismis et al. [62] reported that the majority of blood sources of blood-fed sand flies belonged to the species *Bos taurus*. Karagul and Kasap [29] identified bovine animals, particularly cows, as the most common blood source followed by chickens and goats. Humans were identified as a less common blood source. Among the studied species, *Paraphlebotomus* sand flies were unique in their broader host range, with a particular preference for avian hosts. These results suggest that the host feeding tendency of different sand fly species in Mugla and Aydin provinces are mainly cows, dogs, and chickens, which may be related to the fact that local people keep their animal barns, chicken nests, and their dogs close to their homes. In line with the hypothesis of Dinesh et al. [19], that sand flies tend to feed on cows, cows were the most common source of blood as food source in this study. This may be also related to the higher proportion of cows compared to other hosts in terms of body size, carbon dioxide output level, host-derived volatile organic compounds [7] and other odor secretions. Although the feeding rate from cows is higher than from other hosts, it may not be correct to define this situation as a real host preference because the settlements in the current study are not environments where all hosts coexist [62].

The efficacy of molecular blood-meal identification is contingent upon the quantity of blood ingested and the duration of blood digestion in the midgut of the insect [33]. It is important to note that the quantity of blood ingested by sand flies (1 μl or less) [16] is less than that of mosquitoes (2–6 μl), which is a biological difference that complicates the generalization of results (Clements, 1992). The process of blood digestion can cause DNA denaturation, which can make DNA detection difficult [33, 55], therefore it is essential to use protocols that will detect the minimum amount of DNA. Another factor that must be considered when attempting to identify blood sources using molecular methods is the inhibitory effect of substances found in the tissues of insects, particularly those found in their exoskeleton, head and thorax (Paiva et al. 2007). Substances such as hem in blood [34] can also reduce the efficiency of the PCR reaction. In contrast to molecular methods, conventional serological methodologies for blood meal identification necessitate the production of species-specific antibodies against all potential hosts. These techniques are also constrained by factors such as the unavailability of products for exotic animals, diminished sensitivity, and the possibility of cross-reactivity between species [60]. Despite the limitations and disadvantages inherent to both methods, which are the most frequently employed in blood meal analysis, all samples were successfully analyzed in the present study, and the results obtained by both methods were highly consistent with each other.

Furthermore, determining the susceptibility of sand fly species to specific hosts may hold epidemiological significance in controlling diseases transmitted by vector species. It is useful to ascertain the mammalian host to which a particular species is susceptible, to implement effective measures aimed at reducing host-vector contact. Determining their feeding patterns will facilitate the implementation of protective measures against the diseases caused by sand flies in humans, particularly by identifying the species that tend to feed on human blood [23, 42]. Moreover, a range of control measures, including the use of insecticide-impregnated collars on vertebrate hosts, can be applied to a variety of diseases for which sand flies are vectors.

Although both ELISA and PCR were employed in this study to analyze host feeding patterns, it is worth emphasizing the complementary nature of these methodologies. ELISA offers a cost-effective and relatively rapid means of detecting host-specific antibodies, making it suitable for large-scale screenings. In contrast, PCR provides higher specificity and sensitivity, particularly valuable in cases involving degraded blood meals or mixed-host feedings. The comparative use of these techniques not only enhances the reliability of the findings but also highlights the importance of methodological integration in vector ecology studies. Future research in similar settings may benefit from employing both methods simultaneously to achieve a more robust understanding of host–vector interactions.

While our study provides valuable insights, its design presents several limitations. Sampling was restricted to a narrow two-month temporal window and specific geographical locations within two villages (one per province in Aydın and Muğla). These sites were strategically chosen due to their consistent history of positive sand fly collections and disease endemicity, aiming to maximize the likelihood of obtaining blood-fed specimens. Consequently, our findings represent only a snapshot of sand fly feeding patterns, limiting our ability to capture the full impact of climatic variations, seasonal changes, and diverse habitats on feeding behavior. This restricted coverage means our results do not represent the entire Aegean region and combined with the relatively small sample size of 38 blood-fed specimens, inherently limits the ecological variation captured and the broader spatial generalizability of our conclusions regarding sand fly feeding preferences. Future longitudinal and more geographically expansive studies are needed for a comprehensive understanding.

In conclusion, to the best of our knowledge, this is the first study in the region to analyze ELISA and PCR methodologies in a comparative manner in relation to the host feeding patterns of sand flies. Regardless of the methodology used, the detection of blood meal in field-caught sand flies has the capacity to substantiate the hypothesis that there exists a robust relationship between sand flies in rural areas and reservoir hosts of the diseases they carry. This may facilitate comprehension of the role of endemic domestic animals and further contribute to the planning of strategic control methods by providing a more comprehensive understanding of the eco-epidemiology of vector-borne diseases.

## Supplementary Information

Supplementary Material 1.

## Data Availability

The data sets used and/or analyzed during the current study are available from the corresponding author upon reasonable request.
