# Supplementary material for: Bloodthirsty bites: host-feeding patterns of phlebotomine sand flies from two localities in the Aegean Region of Türkiye
Source: BMC Vet Res. 2025 Jul 25;21:488. doi: 10.1186/s12917-025-04881-y (PMC12291227; doi:10.1186/s12917-025-04881-y)
Supplement: Supplementary file 1 — Supplementary Material 1. [file 12917_2025_4881_MOESM1_ESM.docx]

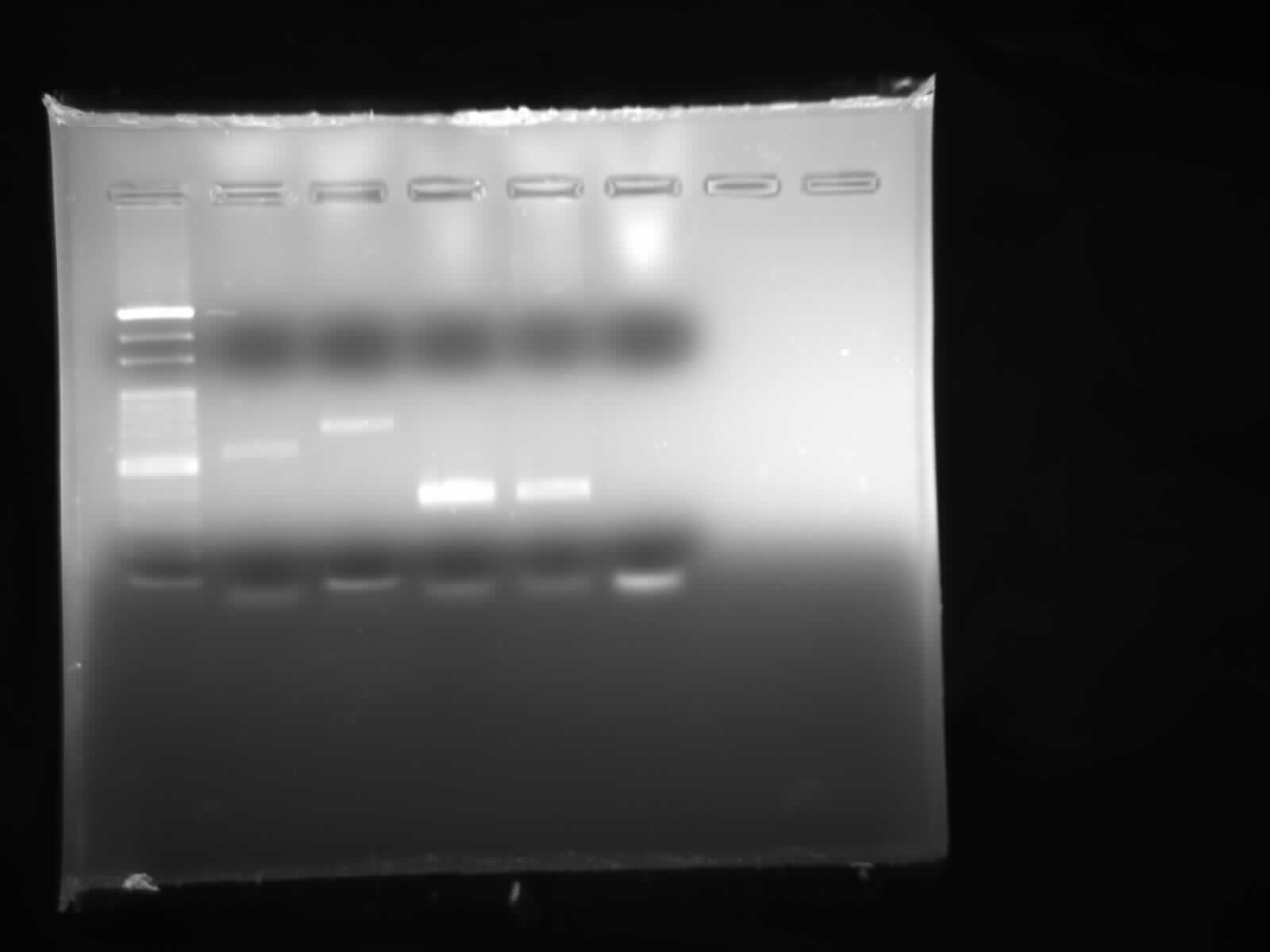


**Figure 1**. Agarose-gel image of possible host’s PCR products (M: marker (100- 1000 bp) band size for **cow:** 561 bp **dog:** 680 bp **human:** 334 bp **chicken:** 383 bp).


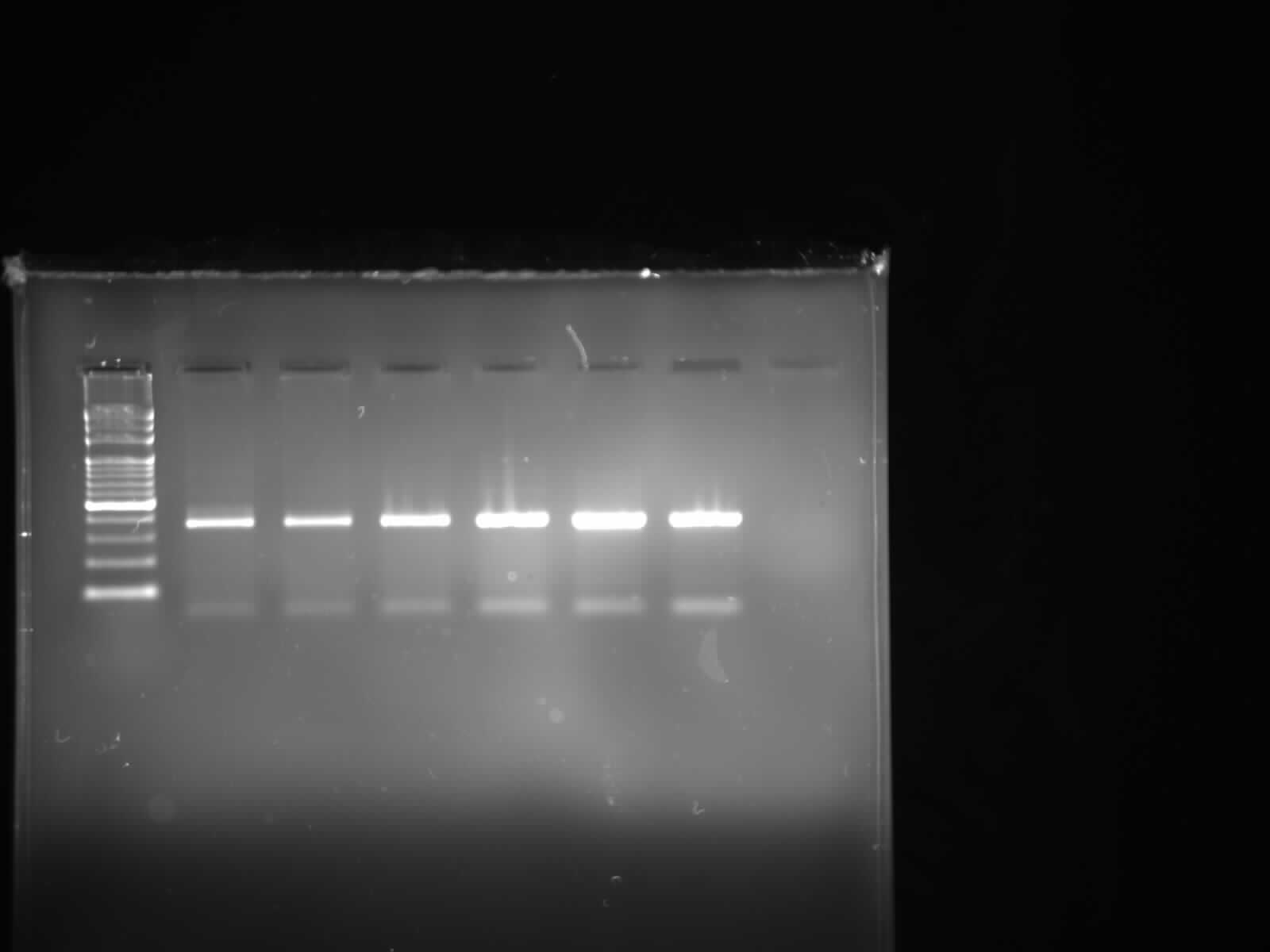


**Figure 2**. Agarose-gel image of PCR products of three sandfly species from Aydin (M: 100 bp, 1-6 chicken PCR prodcuts).
